# Process evaluation of a sport-for-health intervention to prevent smoking amongst primary school children: SmokeFree Sports

**DOI:** 10.1186/s12889-015-1645-1

**Published:** 2015-04-10

**Authors:** Joanne Trigwell, Ciara E McGee, Rebecca C Murphy, Lorna A Porcellato, Michael Ussher, Katy Garnham-Lee, Zoe R Knowles, Lawrence Foweather

**Affiliations:** Centre for Health Promotion Research, Leeds Beckett University, Calverley Building, City Campus, Leeds, LS1 3HE UK; Centre for Public Health, Liverpool John Moores University, Henry Cotton Campus, 15-21 Webster St., Liverpool, L3 2AT UK; Physical Activity Exchange, Research Institute for Sport and Exercise Sciences, Liverpool John Moores University, 62 Great Crosshall Street, Liverpool, L3 2AT UK; Institution of Population Health Research, St George’s, University of London, Cranmer Terrace, London, SW17 0RE UK; School of Sport, Exercise and Health Sciences, Loughborough University, Leicestershire, LE11 3TU UK; Department of Sport and Physical Activity, Edge Hill University, St. Helens Road, Ormskirk, Lancashire L39 4QP UK

**Keywords:** Smoking, Children, School, Intervention, Sport, Process evaluation, Implementation

## Abstract

**Background:**

SmokeFree Sports (SFS) was a multi-component sport-for-health intervention aiming at preventing smoking among nine to ten year old primary school children from North West England. The purpose of this study was to evaluate the process and implementation of SFS, examining intervention reach, dose, fidelity, acceptability and sustainability, in order to understand the feasibility and challenges of delivering such interventions and inform interpretations of intervention effectiveness.

**Methods:**

Process measures included: booking logs, 18 focus groups with children (n = 95), semi-structured interviews with teachers (n = 20) and SFS coaches (n = 7), intervention evaluation questionnaires (completed by children, n = 1097; teachers, n = 50), as well direct observations (by researchers, n = 50 observations) and self-evaluations (completed by teachers, n = 125) of intervention delivery (e.g. length of sessions, implementation of activities as intended, children’s engagement and barriers). Descriptive statistics and thematic analysis were applied to quantitative and qualitative data, respectively.

**Results:**

Overall, SFS reached 30.8% of eligible schools, with 1073 children participating in the intervention (across 32 schools). Thirty-one schools completed the intervention in full. Thirty-three teachers (55% female) and 11 SFS coaches (82% male) attended a bespoke SFS training workshop. Disparities in intervention duration (range = 126 to 201 days), uptake (only 25% of classes received optional intervention components in full), and the extent to which core (mean fidelity score of coaching sessions = 58%) and optional components (no adaptions made = 51% of sessions) were delivered as intended, were apparent. Barriers to intervention delivery included the school setting and children’s behaviour and knowledge. SFS was viewed positively (85% and 82% of children and teachers, respectively, rated SFS five out of five) and recommendations to increase school engagement were provided.

**Conclusion:**

SFS was considered acceptable to children, teachers and coaches. Nevertheless, efforts to enhance intervention reach (at the school level), teachers’ engagement and sustainability must be considered. Variations in dose and fidelity likely reflect challenges associated with complex intervention delivery within school settings and thus a flexible design may be necessary. This study adds to the limited scientific evidence base surrounding sport-for-health interventions and their implementation, and suggests that such interventions offer a promising tool for engaging children in activities which promote their health.

## Background

Smoking is a habit often initiated in childhood, with approximately 207,000 children starting to smoke each year in the UK [1]. Smoking in childhood is a predictive factor for smoking in adulthood [2], and increases the likelihood of early mortality from smoking-related morbidities, including cancer, heart disease and stroke [3,4]. Preventing the uptake of smoking in childhood is an important public health priority [5], with the target of a tobacco free generation by 2025 [6]. Smoking patterns are established prior to experimentation, with the development of attitudes and beliefs [7]. Since one-fifth of children aged 11 to 15 years have tried smoking [8], and a high proportion of nine to ten year old children harbour misconceptions around the harms of smoking [9], it is recognised smoking prevention efforts must target primary school aged children.

Sport-for-health programmes are a growing field in health promotion research, where sport is recognised as an educational platform to support health promotion messages, disease prevention and control efforts [10-13]. Using participatory techniques for delivery, such as active game-based learning and activities with professional athletes, sport-for-health programmes can transmit health prevention messages and change attitudes [14-16]. Moreover, the use of these participatory techniques can aid child engagement with health promotion education [17]. For example, the Grassroot Soccer Foundation delivered a school-based HIV/AIDS education programme to young people in Bulawayo using trained adult football players to educate at-risk youth (7th grade) about HIV/AIDS. Data showed significant improvements in young people’s knowledge, attitudes and perceptions of social support related to HIV/AIDS at post-intervention and five-month follow-up [14]. Furthermore, the Dutch ‘Health Scores!’ programme, combined the use of professional football players as role models with a school based programme to promote a healthy diet and physical activity to socially vulnerable young people (aged 10–14 years). Results demonstrated significant positive intervention effects at four-month follow-up, surrounding self-efficacy for having a daily breakfast and reaching physical activity guidelines, as well as positive attitudes towards vegetable consumption and lower soft drink consumption [15]. In the US and Canada, programmes including Tobacco Free Sports [18], Tobacco Free Athletes [19] and Play, Live, Be Tobacco Free [20] have sought to use sport and coaches to deliver tobacco control interventions. Similarly, in the UK, SmokeFree Sports was piloted in community centres using trained coaches to deliver smoke-free messages to children and young people (7-16 years old) [16,21]. To the authors’ knowledge, impact and process evaluations of programmes using coaches/teachers to deliver smoke free messages via sport in a UK school setting have not been published, highlighting the need for research surrounding sport-for-health interventions and smoking prevention.

SmokeFree Sports (SFS) was a complex, multi-component sport-for-health intervention, aiming to prevent smoking among nine to ten year old primary school children [22]. Compulsory and optional components were delivered by multiple implementers, including SFS coaches and primary school teachers, across 32 interventions schools in the North West of England. This is the first UK based smoking prevention intervention delivered in primary schools of this kind, highlighting the importance of exploring process data surrounding its implementation. Moreover, since sport-for-health interventions are an emergent area of health promotion research where evaluations are sparse and/or have previously lacked scientific rigour [12,23], these interventions would benefit from rigorous evaluations to inform future practice and procedures [11].

Process evaluations are commonly used to measure the extent to which an intervention was delivered or received as planned [24-26], interpret whether it was effective [25,27,28] and indicate its suitability and sustainability for translation into routine practice [29,30]. Informed by process evaluation models [26,27,31], a comprehensive process evaluation was systematically built into the design of the SFS non-randomised controlled trial, examining intervention reach (the proportion of the target audience who received the intervention [27]), dose (the amount of intervention delivered [27]), fidelity (whether the intervention was delivered as intended [27]), acceptability and sustainability. Therefore, the aim of this study was to use collected process data to explore the implementation of SFS from the perspectives of key stakeholders, including children, teachers and coaches. Findings will be used to aid understanding surrounding the feasibility and challenges of delivering sport-for-health interventions, as well as inform interpretations of intervention effectiveness.

## Methods

### Sample and recruitment

In September 2012, primary schools in two local authorities in the Merseyside region of the North West of England were recruited to participate in SFS. Using a quasi-experimental design, schools were clustered into intervention (i.e. schools that received SFS in addition to typical smoking-related education) and comparison groups (i.e. schools that received only typical smoking-related education). The funding agreement required that the intervention was delivered within Liverpool City Council local authority boundaries. Schools situated in Knowsley, an adjacent local authority with similar characteristics to Liverpool in terms of smoking rates (Liverpool: 24.2%; Knowsley: 27.6%) [32], deprivation levels [33] and ethnic composition [34], were used as a comparison group. Notably, Merseyside provides a unique context for the research, as it is home to some of the most deprived local authorities in England [35] where the health of children and young people in Liverpool and Knowsley is worse than the National average [36,37].

All eligible primary schools (i.e. mainstream state schools; n = 154) from both local authorities (Liverpool, n = 104; Knowsley, n = 50) were invited to participate via post and email and received follow-ups via telephone calls. Researchers visited each interested school to share project information with staff acting as study co-coordinators (i.e. class teachers, Head Teachers, Physical Education (PE) and Personal Social Health and Economic (PSHE) Coordinators). Study information sheets and consent forms were given to staff. In total, 43 (27.9% response rate) primary schools agreed to take part in the study, comprising of 32 intervention and 11 comparison schools.

To recruit children, a passive informed consent procedure was used. Parents could opt their child out of the study by signing and returning the opt-out form within a study information pack that was mailed to parents (containing a participant information sheet, parent opt-out form and stamped addressed envelope) or by calling the research team. Schools were visited to obtain child assent, allowing an opt-out timeframe of at least two weeks. Parental consent and child assent were obtained for 1,339 children (96% response rate).

All SFS sport coaches (n = 11), employed through partner organisations [22], as well as teachers from intervention schools involved in SFS (n = 54) were invited to participate in this study. Written informed consent was sought from all parties who agreed to take part.

Ethical approval for the study was granted by Liverpool John Moores University Research Ethics Committee (12/SPS/038).

### SmokeFree Sports

A detailed description of the SFS intervention has been published elsewhere [22,38]. SFS was delivered in primary schools between October 2012 and May 2013, targeting children aged nine to ten years (Year 5). The project was managed by research interventionists at the Physical Activity Exchange at Liverpool John Moores University (LJMU) in a multi-disciplinary partnership with key stakeholders from research, education, public health and sports organisations. Knowledge gained from earlier SFS feasibility studies [16,21,39,40] was instrumental in the evolution of study design, ensuring a ‘bottom-up’ and systematic approach to development as recommended [41].

### Intervention components

Following recommendations from the National Institute for Health and Care Excellence [42], sports coaches and at least one teacher from each participating school were invited to take part in a bespoke training workshop. This comprised a two hour theory and one hour practical, delivered externally during school hours. The workshop provided details of the project and information about smoking, SFS key messages to promote, and practical demonstrations on how to do this via sport. Attendees received SFS training resources, consisting of a training manual and smoke-free pledges for children. The training manual included ten session plans for delivery, designed to cover at least one of the five SFS themes: 1) smoking and health, 2) smoking and sport performance, 3) the contents of a cigarette and financial cost of smoking, 4) smoking and social influences, and 5) the benefits of physical activity. Each session was designed to last for 60 minutes and included a ‘SFS starter’ (one or two warm-up activities), at least one main activity and a cool down. To engage children, each activity was given a child-friendly name (e.g. ‘Nicotine Attack’). Workshops were delivered between October 2012 and February 2013. Teachers completed the training by November 2012 and were instructed to provide feedback to colleagues. Sports coaches received the training prior to visiting schools to deliver SFS coaching sessions.

**Figure 1:**
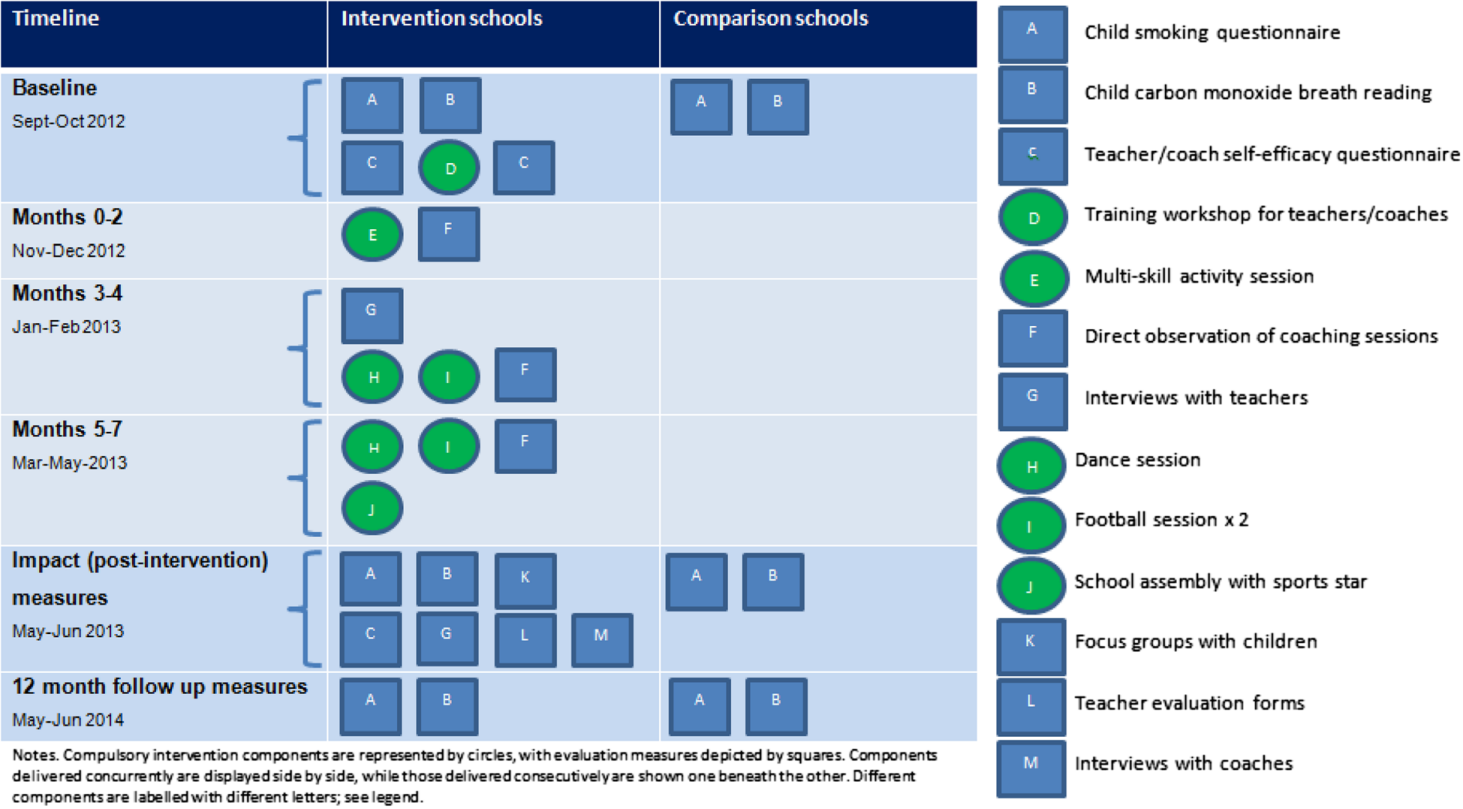
**Schematic overview of SmokeFree Sports intervention.**

SFS coaches delivered five coaching sessions during school hours at each intervention school between October 2012 and April 2013. Typically, SFS coaching sessions replaced usual PE lessons. Schools received one multi-skill, two dance and two football sessions. Teachers (particularly those who delivered PE to Year 5) were actively encouraged to watch/participate in coaching sessions, and incentivised to independently deliver a minimum of five session plans to Year 5 classes over the intervention period. Schools who met this requirement, and completed an evaluation for each session, received SFS-branded sports equipment at the end of the intervention. Teachers were also asked to encourage children to sign the SFS pledge to be smoke-free. On completion of the SFS coaching sessions, a SFS assembly with a local sports star was organised for each school between April and May. A schematic overview of intervention activities is shown in Figure 1.

### Process evaluation

Multiple process measures were integrated into the intervention design, adopting both a progressive and summative approach to data collection in order to obtain the range and depth of data required (see Table 1). Process measures included booking logs, focus groups with children, semi-structured interviews with teachers and SFS coaches, project evaluation questionnaires (for children and teachers), as well as self-evaluations (for teachers) and direct observations (by researchers) of intervention delivery.

**Table 1:** **Data sources used to assess implementation of SFS**

**Data source**	**Sample**	**Date of data collection**	**Implementation aspect assessed**				
			***Reach***	***Dose***	***Fidelity***	***Acceptability***	***Sustainability***
SFS booking logs	32 schools	Oct 2012-Jun 2013	X	X			
Focus groups	95 children (18 focus groups)	Apr 2013-Jun 2013				X	X
Interviews	7 coaches; 20 teachers	Jan-Jun 2013			X	X	X
Self-evaluation of intervention delivery	125 sessions completed by 24 teachers	Oct 2012-Jun 2013		X	X	X	
Direct observations of intervention delivery	50 sessions across 13 intervention schools (10 for each activity type)	Oct 2012-Apr 2013		X	X		
SFS evaluation questionnaire	1097 children; 50 teachers	Apr 2013-Jun 2013		X (teachers only)		X	

Participants in the research study were invited to contribute to the process evaluation. Post-intervention, children across all intervention schools were asked to complete a project evaluation questionnaire. Purposive sampling techniques were employed to select a sub-sample of schools where focus groups and observations of coaching sessions would take place, ensuring schools with one, two and three form classes were represented as well as schools from across each of the five neighbourhood management areas in Liverpool. Each teacher selected a sample of children for the focus groups based on recommendations from previous research (i.e. had the confidence to engage in discussion with the researcher) [43]. Coaches who led SFS sessions were invited to participate in an interview once delivery of their activity type was complete, whilst teachers who delivered SFS were asked to fill-in an evaluation form after each session. Furthermore, using purposive sampling techniques, a sub-sample of teachers who attended the bespoke training workshop were asked to participate in an interview at the end of the study. A sub-sample of teachers who did not attend the bespoke training workshop but delivered PE to Year 5 were also invited to interview. At the end of the intervention all Year 5 teachers and PE deliverers were asked to complete a questionnaire to evaluate SFS.

### SFS booking logs

SFS booking logs were used to assess intervention reach and dose. Throughout the study, the SFS research team recorded demographics (age, sex, ethnicity, highest qualification level, profession, years teaching/coaching experience and smoking status) of those who attended the bespoke training workshop, school details (e.g. class size, deliverer of PE to Year 5) and dates components of the intervention were delivered, including training workshops, coaching sessions and assemblies. Communications (including emails, telephone calls and face-to-face discussions) with teachers regarding the collection of implementation data were also logged.

### Direct observations of intervention delivery

To explore the dose and fidelity of SFS coaching sessions, 50 semi-structured observations of coaching sessions took place (10 for each activity type). Observations were carried out across 13 schools (Alt Valley, n = 4; Liverpool City and North, n = 4; Liverpool South, n = 2; Liverpool East, n = 2; Central, n = 1), with a minimum of two observations overall conducted at each school (mean number of observations at each school = 3.8; range 2 to 8).

One trained researcher was present at each observation and completed an observational record for analysis. These were designed to record session length, class size, teacher presence, as well as details of how the activities were introduced, explained and delivered, children’s engagement and barriers coaches faced. Observation records were first piloted by two researchers and amendments made. Observational records were typed-up following each session for subsequent analysis.

### Self-evaluation of intervention delivery

Self-evaluation of implementation is a common measure of dose, fidelity and acceptability within school-based health promotion studies [44]. To assess implementation of SFS sessions delivered by teachers, teachers were asked to complete an evaluation sheet, using a similar format to that of Operation Smoke Storm [45], immediately following session delivery. Twenty-four teachers completed and returned self-evaluations (n = 125) of intervention delivery.

Self-evaluation sheets were included in the manual and designed to take five minutes to complete. Teachers were asked to score each session they delivered in terms of clarity of instruction given (very easy, minor confusion or major problem), ease of delivering activities (no problem, minor problem, major problem with delivery), adaptions made (no, minor or major adaptions made to the session plan), as well as children’s engagement (easy, minor or major problems for student to engage), understanding (easy, minor, or major problem for students to understand) and enjoyment (all/most students, some, few/no students enjoyed session) of sessions. Teachers had the option to provide additional comments.

### Focus groups

To explore the impact and acceptability of SFS, 18 mixed-sex focus groups with children (n = 95; boys, 45%) were facilitated by a trained researcher. Focus groups comprised of five to six children, lasted between 30 to 50 minutes and were audio recorded using a Dictaphone. Children’s perceptions of smoking, appropriateness of the intervention, and improvements for future implementation were explored. Photographs of SFS games were used to help children recall activity type [46,47]. To aid the credibility of data, facilitators’ reflected interpretations back during the focus groups.

### Interviews

Interviews with teachers and coaches were used to explore the impact and acceptability of the SFS intervention. Twenty teachers (female, 65%; 20–39 years, 85.7%), participated in an interview, including 12 teachers who attended the training (seven with high self-efficacy in delivering SFS post-training, five with low self-efficacy based on self-report questionnaire data (see [38] and Garnham-Lee, unpublished data) and eight who did not. Interviews with teachers took place within two weeks of the intervention ending. Seven (males, 85.7%; 20–39 years, 60%) of the nine coaches who led SFS sessions were also interviewed. Interviews with coaches were conducted face-to-face (n = 6) or via telephone (n = 1) within three weeks of delivery completion.

Semi-structured interview schedules covered all aspects of the SFS intervention, including perceptions of the training, manual, coaching sessions and assembly, as well as their opinions surrounding qualities of SFS deliverers’. In addition, teachers were asked about the delivery of their own sessions, their school’s engagement with SFS and given the opportunity to comment on topics not covered. All interviews were recorded and lasted between 30 and 60 minutes.

### SFS evaluation questionnaires

Post-intervention, children and teachers were asked to complete a SFS evaluation questionnaire designed to assess intervention acceptability and dose (teachers only). In total, 1,097 children (girls, 51%) completed a short evaluation questionnaire comprising of six questions to explore enjoyment, perceived usefulness and general perceptions of the intervention. *Enjoyment of SFS* was assessed using two items, ‘Have you enjoyed taking part in SFS?’ (‘not enjoyed it at all’, ‘enjoyed it a little’, ‘enjoyed it a lot’) and ‘Please give a score out of five for how much you enjoyed each SFS session’ (scored 1 = I did not enjoy the session at all, to 5 = I enjoyed the session a lot). Two items were used to measure *perceived usefulness of SFS*, including ‘Would you recommend SFS to a friend?’ (‘definitely not’, ‘probably not’, ‘probably yes’, ‘definitely yes’) and ‘How useful is SFS in helping you or other children to stay smoke-free (to not smoke)?’ (‘not at all useful’, ‘fairly useful’, ‘very useful’). *General perceptions of SFS* were explored by asking, ‘On a scale of one to five, how would you rate SFS?’ (1 = very bad, 3 = OK, 5 = very good) and providing an open text box for additional comments.

For teachers, a one-page evaluation questionnaire was designed to collect information about smoking education delivered to Year 5 children across the 2012/13 academic year, delivery of SFS pledges as well as explore teachers’ perceptions surrounding the acceptability of SFS. Questions assessing acceptability of SFS related to perceived usefulness, strengths and weaknesses as well as general views of the intervention. *Perceived usefulness of SFS* was assessed using two items, ‘How useful do you think SFS will be in helping children to stay smoke-free?’ (‘not at all useful’, ‘fairly useful’, ‘very useful’) and ‘Would you recommend SFS to other schools?’ (‘definitely not’, ‘probably not’, ‘probably yes’, ‘definitely yes’). To explore intervention strengths and weaknesses, teachers were asked to list three things they ‘liked most’ about SFS and three things they would ‘improve’. *General perceptions of SFS* were explored by asking, ‘On a scale of 1 to 5 (poor to excellent), how would you rate SFS?’ and providing an open text box for general comments. Fifty teachers (females, 62%) filled in a questionnaire post-intervention.

### Data preparation and analysis

SFS booking logs were maintained and analysed in Excel. Quantitative data collected via self-evaluations and semi-structured observations of delivery were coded and inputted into SPSS Version 20 and descriptive statistics generated. Direct observational data was coded on a three point scale (options: *yes, in part, no*). Sessions were divided into the following sections *introduction, warm-up, main section* and *cool down* for coding, with each activity within the sections scored separately. Sections were scored against the criteria listed in Table 2.

**Table 2:** **Direct observations coding framework**

***Introduction***	*Did the coach introduce themselves and the SFS intervention?*
***Warm up and main section***	*Was each activity delivered as outlined in the manual?*
	*Was the name of the game cited and the purpose of the activity explained as outlined in the manual?*
	*Was key message # delivered as outlined in the manual?* (item repeated for each message outlined for delivery)
***Cool down***	*Was the activity delivered as outlined in the manual?*
	*Was key message # delivered as outlined in the manual?* (item repeated for each message outlined for delivery)

To aid the reliability of data, a sub-sample of fidelity scores were cross-checked by a second researcher; inter-coder reliability was high. Total scores were calculated for each session and converted into a percentage for comparisons across activities ((total fidelity score across components of observation ÷ number of components for session type) × 100). Fidelity was scored as low (≤33%), average (34-66%) or high (≥67%), as categorised in previous research [48]. For fidelity to be defined as acceptable (high), at least two thirds (67%) of the session had to be delivered as intended.

All focus group and interview recordings were transcribed verbatim for analysis. Transcripts as well as open responses from self-evaluations of intervention delivery were imported into NVivo version 10 and subjected to thematic analysis [49]. This process involved reading and re-reading text and assigning broad thematic codes, some of which were pre-defined from topics covered in the group schedule. Subsequently, broad codes were collapsed into higher and lower order themes and descriptive and interpretive summaries were written based on recursive engagement with the data. A combination of inductive analysis and deductive techniques were used to generate codes. To aid the credibility and trustworthiness of the results, analyses and interpretations of the data were discussed and checked with the research team and amendments were made. The use of a mixed methods approach allowed for the conformability of data through the process of triangulation [50].

## Results

### Reach

Overall, the project reached 30.8% of eligible schools (32 out of 104). A number of schools provided reasons for declining to take part (including being too busy, Year 5 teacher on sick leave, new teacher coming into post, and already being in receipt of external projects), whilst others did not reply to recruitment literature or return telephone calls and therefore reasons for non-participation are unknown.

From these 32 schools (including 45 Year 5 classes), 1073 children received components of the SFS intervention. Thirty-one schools (44 classes) completed the SFS intervention (school attrition rate, 3.1%). Completion was defined as at least one teacher attending the bespoke training and each Year 5 class receiving five SFS coaching sessions and a SFS assembly. One school withdrew during the study period citing school staffing issues. Schools that received the full intervention were dispersed across all five Neighbourhood Management Areas in Liverpool (Alt Valley (n = 8), Liverpool City and North (n = 7), Liverpool South (n = 7), Liverpool East (n = 7) and Central (n = 2)). Three-quarters of these schools were located in the 10% most deprived Super Output Area’s in England [51].

The bespoke training was attended by 33 teachers (from 32 schools; job roles including: teacher, n = 25; teaching assistant/learning mentor, n = 6; sports coach, n = 2) and all SFS sports coaches delivering sessions (n = 11). Teachers (54.5% female; 62.5% aged 20–39 years) who attended the training had between one and 34 years of coaching or teaching experience (mean = 9.7 years, s.d. = 7.5). Four teachers reported to currently smoke. Coaches (81.8% male; 72.7% aged 20–39 years) had between two and ten years of coaching experience (mean = 3.3 years, s.d. = 1.1). All coaches reported not to smoke.

### Dose

Across the course of the intervention period, 223 out of the planned 225 SFS coaching sessions (each class expected to receive five SFS coaching sessions) and 31 SFS assemblies were delivered. On average, a 29.5 day interval occurred between coaching sessions (range, 0 to 90 days, s.d. = 22.4). Observational data revealed the duration of coaching sessions ranged from approximately 30 to 60 minutes (approximate mean = 48.1 minutes, s.d. = 8) (reasons for disparities in length of coaching sessions are discussed under ‘Fidelity’). Assemblies lasted between 15 and 30 minutes based on time allocated by schools. Reasons for variations in assembly duration included length of time during which the SFS sports star discussed his/her sporting achievements and time allocated for questions. Overall, duration of the SFS intervention ranged from 126 to 201 days (mean = 169.4 days, s.d. = 21.5).

In total, teachers reported that they delivered 125 SFS sessions, with 56.8% of classes receiving at least two sessions and nearly half (47.5%) of classes receiving a minimum of five. Data from teachers’ SFS evaluation questionnaires revealed 20 Year 5 classes signed a SFS pledge (43.5%, approximately 470 children). Eleven Year 5 classes (25%) received the SFS optional intervention components in full (i.e. received a minimum of five SFS sessions from teachers and signed the SFS pledge).

Fifteen schools who participated for the study’s duration did not return completed evaluations for all Year 5 classes within their school (in two schools with multiple Year 5 classes, sessions were only delivered in one Year 5 class). Reasons recorded by the research team for non-delivery/non-completion of evaluations in hierarchal order included, misplacing training manual, lack of time to complete session evaluations, extended period of sick leave taken during intervention period or Year 5 teacher/PE teacher entered post part-way through school year. Despite repeated attempts by the research team to contact teachers, reasons for non-delivery or completion of evaluations are unknown for nine Year 5 classes.

### Fidelity

Direct observational records were utilised to score the fidelity of 50 SFS coaching sessions. Overall, the average fidelity score for SFS coaching sessions was 57.8% (range 30.5 to 92.1%, s.d. = 15.8). Whilst 28% of sessions observed scored high for fidelity (67 to 100%), a further 70% were recorded as average (34 to 66%). Mean fidelity scores differed across session type (session 1 = 72.9%; 2 = 56.1%; 3 = 52.2%; 4 = 55%; 5 = 58.2%).

Reasons for disparities in the fidelity of SFS coaching sessions were explored further during interviews. Coaches recognised the importance of consistency in adhering to session plans but identified a number of barriers to delivering sessions as intended (see Table 3). Barriers related to the school settings and children, with the former leading to more frequent deviations from session plans. In regards to the school setting, barriers included class size (too many or too few), limited time relating to organisation (late arrival of class, disruptions in hall leading to early finishes) and the environment (hall size, delivering outside due to no access to sports hall). Furthermore, coaches reported modifications were sometimes made to sessions based on children’s behaviour and diverse physical abilities, and that delivery of sessions improved over the course of the intervention period as familiarity with activities and messages increased.

“I think the first 10 schools weren’t as good as the last 20 schools, purely because it was, it was something new you hadn’t done it before. We had delivered the games before but trying to get your messages in, and they weren’t fluent, the last sort of, I’d say the last two thirds of the sessions were so fluent because we’d run through it”. (Coach 1, interview data)

**Table 3:** **Barriers coaches faced in delivering SFS sessions**

**Barriers to delivery**	**Quotes**
***School setting***	
*Class size*	*“Like the one [name of school], they’ve only got like nine kids in each class, so we delivered with nine kids. So obviously the session changes, we ended up putting an extra game in I think there, just because you go through things too quickly”.* (Coach 1, interview data)
*Environment*	*“Obviously some schools have a big hall and some schools don’t have such a big hall, so it was mainly the facility we could use and also class size that altered on how the session was delivered”.* (Coach 2, interview data)
*Time*	*“Yeah, we found that when we were going after dinner time, so it was normally the half one session. Obviously the children had just got in from dinner time so where the session was meant to start at half one, by the time they have gone back up to the classroom, got settled, got changed that might have went to a quarter to two and obviously you have to wrap that back up and have the session done for maybe twenty, twenty five past [two] or so”.* (Coach 5, interview data)
***Children***	
*Behaviour*	*“… I had to adapt that in a couple of schools because they [children] were just getting silly and trying to hit each other really as they were coming through, so I adapted that slightly”.* (Coach 7, interview data)
*Disabilities*	*“The only one [session] we had to modify… there was a few kids with disabilities in the school, in the class that we done, and that was just stuff we know how to adapt to anyway”.* (Coach 6, interview data)

Notably, barriers reported by coaches to intervention fidelity were supported by direct observational data.

To determine fidelity of SFS sessions delivered by teachers, self-evaluation data was used. For 50.8% of the SFS sessions led by teachers no adaptions were reported, a further 43.5% of sessions were delivered with minor amendments. Data from self-evaluations revealed, 91.9% of sessions that took place were deemed *‘easy to deliver’*, with a further 87.1% delivered with *‘no problems experienced’*.

Self-evaluation forms requested explanations for any modifications made to session delivery. Reasons were explored further during interviews (12 out of the 20 teachers interviewed reported to deliver SFS sessions). A summary of barriers faced by teachers in the delivery of their sessions impacting on intervention fidelity can be found in Table 4. They include: time restrictions, environment*,* children’s educational understanding, managing children’s challenging behaviour when on the ‘smoking’ team and children’s preferences for an activity.

**Table 4:** **Barriers teachers faced in delivering SFS sessions**

**Barriers to delivery**	**Quotes**
Time	*“Did not have time to complete all activities”.* (Teacher, school 20, self-evaluation data)
Environment	*“It was weather more than anything you know. The game where we had to have the cones and you had to turn them over, we were out in a force 10 gale and they were just blowing everywhere and they were getting really upset”.* (Teacher, school 1, interview data)
Educational understanding	*“Some children didn’t have good knowledge of human body - this meant that they needed lots of support with bean bag game”.* (Teacher (1), school 41, self-evaluation data)
Behaviour	*“I quite often had characters in a bit of a sulk because of it [being put on the ‘smokers’ team]”.* (Teacher, school 13, interview data)
Children’s preferences	*“Used the same messages but changed the sport to basketball instead of football due to previous issues with some of the girls engaging with the context. The children loved the session”.* (Teacher (1), school 8, self-evaluation data)

### Acceptability

Overall, SFS was viewed positively and considered by teachers to align with the PSHE curricula.

“A very valuable programme that has supported our PSHE curriculum in school”. (Teacher, school 24, SFS evaluation questionnaire data)

Questionnaire data revealed almost all children enjoyed taking part in SFS (98.5%), with 85.1% of children scoring SFS five out of five. Furthermore, 96.8% of children reported they would recommend the intervention to a friend, and 88% considered SFS ‘very useful’ in helping them to stay smoke-free.

Similarly, 82% of teachers scored SFS five out of five. All teachers stated they would recommend SFS to other schools and 80% thought SFS would be ‘very useful’ in helping children to stay smoke-free. In addition, coaches and teachers praised the organisation of the intervention and professionalism of staff.

“I think everything is set up well, it’s well organised, it’s well run, the messages are clear and concise … you know everything is in place for it to be successful”. (Coach 1, interview data)

“A really excellent planned and delivered programme with enthusiastic and committed staff”. (Teacher, school 3, SFS evaluation questionnaire data)

### Physical activity as a vehicle for delivering smoking education

Collectively, children, teachers and coaches viewed physical activity as a useful mechanism to engage children in smoking prevention education.

“Like when you’re in class and your teacher’s telling you not to smoke and you’re sitting there going ‘I’m bored’, [and they are saying] ‘like no don’t smoke and it’s bad for you’, and you’re just sitting on the carpet….what we do every day and we’re thinking this is just a boring lesson…. and then the coaches are better ‘cause as [child] said they do loads of activities with you… they try and make it as fun as possible and then that’s why I like SFS coming in”. (Girl, school 3, focus group data)

In particular, children and teachers felt SFS offered a *“fun”* learning experience where smoking-related messages were demonstrated and ‘experienced’ through physical activity, thus aiding children’s understanding.

“They taught us through fun and games and using sport to help us understand how with the football, if they use the footballs to go down your throat [dribbled ball through cones], and how hard it was if you smoke, and if you don’t smoke how it was easier”. (Boy, school 38 (gp 3), focus group data)

“Instead of them being told that information and writing it down they can actually feel the effects on their body which is they learn from experience so it’ll be more vital to them in their understanding”. (Teacher (1), school 2, interview data)

Similarly, coaches regarded physical activity as a useful vehicle for delivering smoke-free messages due to the inherent relationship between physical activity and smoking as well as children’s interest in the pastime.

“I think personally football is the best thing to use [to deliver smoke-free messages] because in football… if you go to young children ‘who wants to be footballers?’ and you tell them about like they [footballers] are training every day and they are not smoking, the children are going to want to look up to… so to use football as a way to get them away from smoking I think it is the best method”. (Coach 3, interview data)

This method of delivery was considered *“inclusive”* by coaches and teachers, and according to coaches since children enjoyed and were familiar with physical activity, this would also encourage participation in SFS.

### Perceptions of the SFS bespoke training workshop and manual

Collectively, teachers and coaches viewed the training and manual positively. Two teachers did however feel the training was unnecessary when coupled with the manual.

“I think I could have got by without it because as I say you see it again and I think this book [the manual] by the way was very helpful”. (Teacher, school 1, interview data)

Whilst teachers and coaches valued the importance of the theoretical and practical sessions of the workshop, it was felt the practical session worked particularly well in preparing for the delivery of SFS.

“I thought it was good the way we got it from other people because you are seeing people who have done this before so you know what is expected then”. (Coach 3, interview data)

Moreover, teachers reported the manual aided the delivery of their sessions, praising the clarity of the instructions and simplicity of the session plans.

“The manual, I thought was really useful, it breaks down [activities] really simply with clear explanations”. (Teacher, school 15, interview data)

Coaches also recognised the importance of the manual, using it to refresh their knowledge of activities and key messages to deliver. Recommendations to improve the training and manual were offered. In relation to the practical element of the training, teachers and coaches felt more time to practice delivery would have been beneficial. For the theory session, teachers felt this section could have been condensed, whilst coaches reported more interactive tasks would have been beneficial. Coaches also suggested that information surrounding potential issues that children may raise about smoking and how this could be addressed warranted attention in the training and/or manual. Coaches and teachers thought the usability of the SFS training manual could also be improved through the inclusion of visual diagrams and/ or a DVD of activities.

### Coaching sessions and assembly

Questionnaire data revealed the majority of children enjoyed the SFS coaching sessions and assembly; 71.4% of children reported to enjoy the multi-skill session ‘a lot’, 67.2% the dance sessions, 68.7% the football sessions and 72.2% the assembly.

During focus groups, children stated they enjoyed SFS coaching session because of the games played and were able to describe elements of favourite activities. Moreover, sessions were considered fun, educational and offered children the opportunity to experience different activities. In general, teachers and coaches gave a positive overview of the coaching sessions, commenting that children appeared to enjoy the sessions, showed enthusiasm to partake in games, and were responsive to the smoke-free messages, answering and asking coaches’ questions (see Table 5 for a summary of positive aspects of coaching sessions).

**Table 5:** **Positive aspects of coaching sessions**

**Positive aspects of coaching sessions**	**Quote**
Fun activities/enjoyment	*“I enjoyed the Liverpool coaching and activity where they did all the football with you”.* (Girl, school 38 (gp 1), focus group data)
	*“…the whole noise, the kids laughing, joking and at the end of the session when you are doing the feedback and the Q&A’s, they knew all the answers, they had remembered all the things”.* (Coach 2, interview data)
Educational/engaging	*“My favourite one [game] was ‘Smoking Fools and Cool Dudes’ because it shows how much harder it was for a smoker to catch up with the non-smokers”.* (Boy, school 11 (gp 2), focus group data)
	*“The participation was really good, they really enjoyed it, there was no one that didn’t want to take part, and they were answering questions very well, so they were well engaged in the lessons”.* (Teacher, school 36, interview data)
	*“Really well I thought it was well received by the staff. The kids loved it and I know myself and the other coach really enjoyed delivering it and just from the feedback and the questions we asked at the end of each session they were aware of all the messages we wanted to get across within the sessions”.* (Coach 2, interview data)
Experience different activities	*“I like it [dance], and I’ve never really had the opportunity to like to it and it’s unusual to get things like that”.* (Boy, school 3, focus group data)

Coaches noted that on occasion smoke-free messages were met with resistance or confusion; coaches were however confident in addressing these issues with children.

“Well at the start [of the session] you seem to get a little mix [in response to messages] because you would get people who would say ‘my mum smokes and she still goes the gym and that’, so you would say ‘do you think if she didn’t smoke and she went the gym she would be a lot healthier or maybe able to go the gym a bit more?’”. (Coach 3, interview data)

Despite an overall positive review, negative aspects or types of coaching sessions were reported (see Table 6 for details). Predominately, children stated individual preferences for an activity and disliking others. Specifically, children found often one football session to be less favourable than another based on the football team the coaches represented.

“I only said I didn’t like the Liverpool one [football session] is because I do not like Liverpool football team”. (Boy, school 18, SFS evaluation questionnaire data)

**Table 6:** **Negative aspects of coaching sessions**

**Negative aspects of coaching sessions**	**Quote**
Session type/activities	*“It was like embarrassing and I’m not good at dance”.* (Boy, school 16 (gp 2), focus group data)
Unfairness of games	*“The only thing the children didn’t enjoy at first was the unfairness, what they perceived to be unfair by not having the same chance as the other ones [on the non-smoking team]”.* (Teacher 1, interview data)
Repetitive nature of sessions	*“I understand the use of repeating activities but I felt that they found it slightly boring…”* (Teacher (1), school 2, interview data)
Messages delivered were perceived to be incorrect	*“The [football] coach got things technically wrong, he used words like ‘plaque’ instead of ‘phlegm’ and other things like statistics he got wrong”.* (Teacher (3), school 2, interview data)
Lack of clarity of message/purpose of game	*“Some more [sessions] than others, the football were set out really well with the representation … But it wasn’t quite as clear [the purpose of the activity] say in the dancing”. (Teacher (1), school 38, interview data)*
Sedentary nature of games	*“I didn’t like it when you had to sit down and write because it wasn’t really active”.* (Boy, school 27, focus group data)
	*“I find it important to get them straight into it [the activity] and I think the dance did that whereas the football maybe could’ve said half of what he said”.* (Teacher 18, interview data)
	*“…back to the warm up you know more kids, instead of like standing at the cones at the end, maybe like setting them a different challenge while they are waiting round because obviously the only people that were working were in the middle…”.* (Coach 4, interview data)

Negative aspects of coaching sessions were discussed in more detail by coaches and teachers. The ‘unfairness’ of being hindered when on the smoking team was considered by teachers as an aspect of the sessions that children sometimes did not enjoy. One teacher also reported they felt children found the sessions un-stimulating due to their repetitive nature, a view not shared by children.

A further criticism of the programme, raised by children, teachers and coaches, related to sessions having extended periods of sedentary time. Sedentary periods were attributed to having large groups with multiple children on the same task and, spending too much time talking through messages rather than demonstrating these through activities.

In relation to messages delivered, limitations were discussed. Teachers noted that coaches sometimes provided children with information that was “*technically wrong”* and believed it was essential coaches had a full understanding of messages before delivery of sessions. It was also recognised that the clarity of messages and purposes of games delivered could be improved in particular sessions. Additional recommendations surrounded utilising more visual aids to reinforce smoking messages and having a greater focus on assisting children to deal with peer pressure.

Regarding the assembly, children were able to recall the assembly and enjoyed seeing visual resources, listening to SFS sports stars as well as asking questions, receiving certificates and autographs. Overall, teachers viewed the assembly in a positive light and an appropriate way to end the project; the SFS assembly was deemed a *“highlight”* of the intervention and SFS sports stars considered *“inspirational”.*

“.. excellent [the SFS assembly], no I thought that bringing the people in [SFS sports star] just gave another message again…. we can stand there till we’re blue in the face saying ‘don’t smoke and this and that’ but to have somebody who’s been successful in a sporting field, I think it just notches it up even more doesn’t it?” (Teacher (2), school 16, interview data)

### Teachers’ sessions

From the collective viewpoints of teachers who completed self-evaluations and/or participated in interviews, data revealed teachers own delivery of SFS was positive. Data from session evaluations revealed sessions were easy for children to engage in (84.7%) and understand (85.5%), and that most children appeared to enjoy the sessions (92.7%).

“Children were exhausted! Messages understood. Good for general fitness, will do this again. Felt pupils engaged in sessions”. (Teacher, school 27, session plan evaluation)

Moreover, it was also noted that conveying SFS messages to children worked better than expected, sessions linked well with the curriculum and led to additional class work

“The talking bits worked a lot better than I expected”. (Teacher, school 1, interview data)

“Enjoyable activity that actually led to a lot of class work where children were amazed at the cost of smoking!” (Teacher, school 12, session plan evaluation)

During focus groups, whilst some children were able to recall teachers delivering SFS sessions and discussed various games played, most groups were unable to remember whether activities were played or discussed games that were not recognisable from SFS session plans.

### Deliverer of SFS

Children, teachers and coaches were asked about the qualities of deliverers. Whilst strengths of teachers and coaches delivering SFS sessions were recognised (see Table 7 for a summary of advantages of using teachers, and Table 8 for coaches, to deliver SFS), disadvantages of deliverers were also discussed.

**Table 7:** **Advantages of using teachers to deliver SFS**

**Strength of teachers**	**Quote**
**Children’s perspectives**	
Respected	*“Teachers, because they can get our attention easily and we have to listen”.* (Boy, school 38 (gp 2), focus group data)
Knowledge of smoking issues	*“They understand it [smoking issues] more”.* (Girl, school 18, focus group data)
Experience of working with children	*“Because they’re trained to be with children and teach children”.* (Boy, school 18 (gp 2), focus group data)
Relationship with teacher	“*We all know the teacher and trust the teachers more”.* (Boy, school 8 (gp 1), focus group data)
**Teachers’ perspectives**	
Relationship with child	*“I know the kids so I can look ahead and see which activities they might struggle with”.* (Teacher, school 13, interview data)
**Coaches’ perspectives**	
Relationship with child	*“Obviously they work with those children everyday so obviously they know what makes the kids click”.* (Coach 4, interview data)
Time to follow-up messages	*“If they get into it they can deliver these messages constantly, you know five days a week with the kids”.* (Coach 1, interview data)

**Table 8:** **Advantages of using coaches to deliver SFS**

**Strength of coaches**	**Quotes**
**Children’s perspectives**	
Role model	*“Because they don’t smoke and they’ve teached us not to smoke when we’re older so we can be like them and enjoy sport in our lives”.* (Girl, school 4, focus group data)
Fun	*“Because they [coaches] were like fun”.* (Boy, school 3, focus group data)
Experience and knowledge	*“They [coaches] know more about smoking and sports than teachers do”.* (Boy, school 20, focus group data)
**Teachers’ perspectives**	
Knowledge of SFS/experience of delivery	*“They [the coaches] know the whole project and the programme inside out and back to front”.* (Teacher (2), school 16, interview data)
Fresh approach	*“It’s good for the kids to have coaches coming in and getting fresh ideas and ways of looking at things”.* (Teacher (2), school 5, interview data)
Authority and credibility	*“When a coach comes in especially when they’ve got the Liverpool or Everton badge they think they’re professionals and they can have almost more authority and credibility over the kids”.* (Teacher, school 15, interview data)
**Coaches’ perspectives**	
Coaching experience	*“We are more, for our job, specialised in the sport element”.* (Coach 5, interview data)
Experience delivering SFS	*“…maybe a bit more knowledge of the sessions and the drills themselves, so how to set them up and when to break things down to say when to get the messages in”.* (Coach 4, interview data)
Role model status	*“…a little role model to look up to because we made the sessions fun and made them enjoy it whether they support Liverpool or Everton or not to being more beneficial”.* (Coach 6, interview data)
Belief and enthusiasm	*“I actually had a belief in what I was saying, it gives more belief to your sessions, it gives you more clarity, a better underpinning of it so you’re not just basing it on what it says, your basing it on what you think and what you believe and what you know and then all of a sudden it’s got more integrity”.* (Coach 1, interview data)
Novelty factor	*“I’d say sports coaches are like adored in many aspects, especially like you know, it’s a fresh face”.* (Coach 1, interview data)
Power of football badge	*“As a football coach coming into the children I think that when they see us they don’t see just a normal person they see Everton Football Club and they see all their idols who play for that team or the people they look up to, so that when you start delivering the SmokeFree sessions they listen because they think about how Fellaini or Pienaar have listened to their coach”.* (Coach 3, interview data)

Notably, coaches felt teachers often lacked enthusiasm for physical activity and confidence in delivering PE as well as concerns surrounding teachers’ smoking status.

“I bring enthusiasm which a lot of teachers lack enthusiasm for the actual sport side, they aren’t particular fond of doing PE they see it as a… like a… a time of the week were they don’t really won’t to do it but they have to it…I think I also bring belief in the project whereas you know there are teachers from certain schools that you saw having cigarettes in between sessions or coming in from sessions having been on their cigarette break”. (Coach 1, interview data)

This latter concern was reiterated by children as a disadvantage of using teachers, impacting on their credibility when discussing the importance of being smoke-free.

“I'd say it’s bad, because if some of the teachers smoke and they have to deliver things about how bad smoking is, then instead of discouraging them about smoking they could be encouraging them”. (Boy, school 38 (gp 2), focus group data)

The only disadvantage stated by children to the use of coaches related to their lack of relationship.

“’Cause we didn’t really know their name but we know [teacher’s name] better but we didn’t know them much”. (Girl, school 16 (gp 2), focus group data)

Moreover, teachers and coaches recognised the benefits of combined delivery, stating teachers could learn from coaches.

“I think the coaches help the teachers to show them because they’ve been trained in it”. (Teacher, school 30, interview data)

“The teachers that got up and got involved and took part [in coaching sessions] enjoyed it and got a lot out of it and seen how we delivered it, because I think a lot of them were like I’m not too sure how to do it but hopefully we gave them ideas and confidence to say ‘here’s some ideas, deliver it this way’”. (Coach 2, interview data)

In reality, however, coaches noted that whilst teachers were present for the majority of sessions, teachers’ engagement with coaching sessions ranged extensively, from sitting in the sessions marking work, assisting with behavioural issues and activities, to actively participating in the session with children.

“They were asking what they can do… joining in …we went to [name of school], we did the same for three classes in [name of school], and the young teaching student… he joined in, he came down every week to help out because he enjoyed the sessions”. (Coach 1, interview data)

“But I did try and say you know, they need to be part of it, some of them would but most of them didn’t stay or even watch or couldn’t really”. (Coach 7, interview data)

Combined delivery was also considered by coaches and teachers to provide variety in delivery, with coaches recognising teachers could reinforce messages delivered during coaching sessions.

### Sustainability

Firstly, sustainability of SFS was discussed in relation to the importance of effect maintenance. Teachers and coaches recognised the importance of maintaining intervention delivery within the school setting to ensure the intervention had a long-term impact on non-smoking behaviour.

“It’s effective now… but I feel if it doesn’t continue they’ll just get pressured anyway with peer pressure”. (Teacher (1), school 2, interview data)

Secondly, teachers felt a wider cultural awareness of SFS was needed across the school, in order to aid intervention sustainability. As a minimum, this involved informing all staff and children about the nature of the SFS study. Wider engagement of staff and children was recommended, with teachers suggesting enrolling more staff on the bespoke training or providing in-house staff training (allowing for greater attendance), feeding back training to all staff members and engaging all year groups in the intervention.

Coaches were in agreement with teachers, recognising the importance of training more staff members in order to engage them in the intervention and recommending the intervention target additional year groups to aid cultural awareness of SFS within the school environment.

“I think when we do the training… we should have a few more of the teachers present because the teachers didn’t actually realise actually what we were doing”. (Coach 6, interview data)

Crucially, in general, children, teachers and coaches were in support of participating in future SFS interventions.

## Discussion

This study aimed to evaluate the process and implementation of SFS, an innovative multi-component sport-for-health intervention to prevent smoking among nine to ten year old primary school children from North West England, from the perspectives of multiple stakeholder groups. Data showed that whilst intervention reach (at the participant level) was high, disparities in dose and fidelity were apparent. These findings are consistent with other school-based interventions [29,52,53] and likely represent the challenges of implementing health promotion activities across multiple school settings. Nevertheless, SFS was considered acceptable and valuable insights for improved sustainability were offered. This study adds to the limited scientific evidence base surrounding sport-for-health interventions and their implementation [12], and suggests that such interventions offer a promising tool for engaging children in activities which promote their health.

SFS reached 30.8% of eligible schools, with various reasons given for non-participation. The number of intervention schools to participate in the study are similar to other UK school-based smoking prevention studies [54,55], however, it should be recognised sampling techniques employed varied. Notwithstanding this finding, data showed intervention reach at the participant level was high, with more than 1000 children taking part in SFS. This is likely to be attributable to intervention delivery via a school setting and how SFS was accommodated within the school timetable, often a substitute for usual PE lessons and thus participation was compulsory. In comparison, participation rates for the SFS community pilot were lower [16], which could be due to the smaller reach of community centres in relation to primary schools and voluntary involvement in activities. Importantly, low attrition rates were observed, and comparable to UK large scale secondary school-based smoking prevention studies prior to follow-up periods [54,56,57].

Disparities in intervention dose were apparent, and related to optional components of the intervention, including additional delivery of SFS sessions by teachers and signing SFS pledges. Optional sessions were received by 56.8% of Year 5 classes, with 47.7% having the recommended five extra sessions to reinforce the SFS key messages. SFS pledges were signed by nearly half of Year 5 classes. Despite a generous scheme to incentivise teachers to deliver optional components, results indicate a lack of engagement amongst some teachers, which may have implications for the effectiveness and sustainability of SFS.

Variations in teacher engagement may be a result of delivering the intervention in the school setting. Previous school-based physical activity intervention studies have found similar factors have impacted on delivery, including the time required for intervention implementation and the completion of research logs [58], the use of substitute teachers [59], and lack of equipment and facilities [60].

An acceptable level of fidelity (defined as ≥67% of the session delivered as intended) was found in 28% of SFS coaching sessions, whilst adaptions were made to nearly half of the sessions that were delivered by teachers. Variations in dose and fidelity have been observed in other health promotion interventions within school settings [48,53,57]. Consideration must be given, however, to how dose and fidelity are measured and scored across studies as well as the amount of flexibility that is incorporated into the design of the intervention (e.g. classifications for an acceptable level of fidelity).

Whilst SFS session plans were designed to be pragmatic for consistent implementation across schools, several barriers to delivering sessions as intended were cited by coaches and teachers. Barriers to intervention fidelity (e.g. environment, class size and children’s physical disabilities) related specifically to the use of physical activity as a vehicle for delivering smoking prevention education. In relation, barriers reported regarding children’s engagement and school settings suggest general adaptions to the session plans are necessary to aid implementation of the intervention (e.g. reducing session length). Moreover, session plans were adapted to aid children’s educational understanding and participation, as well as meet their preferences, a practice that is recommended in school-based health promotion [53], to promote intervention ownership and children’s engagement [44], ensuring a child-centred ethos. It is therefore suggested that greater flexibility in the design of session plans is needed to ensure fidelity of intervention implementation is not compromised. The ASSIST study has previously documented success in integrating such an approach, where a ‘traffic light system’ was designed labelling intervention components as red (essential component of the intervention and should not be omitted), amber (intervention component intended to consolidate skills and can be omitted during particular circumstances such as serious time constraints) and green (this is a linking activity and can be omitted if there are time constraints) [53]. Before flexibility is built into the design of SFS session plans, further research is needed to investigate the impact of individual components and explore conditions in which modifications to the intervention should be made [44].

Overall, children, teachers and coaches considered SFS to be an acceptable intervention to educate children about smoking. The intervention was praised by teachers and coaches for its organisation and professionalism and described as engaging, fun and educational. Moreover, similar to the SFS feasibility studies [16,39,40], almost all children reported enjoying taking part in SFS, with more than 80% of children and teachers rating the intervention five out of five. Collectively, children, teachers and coaches generally found intervention components useful and deliverers’ viewed session delivery as ‘easy’. It is important to note, however, variations in fidelity of sessions and dose delivered were observed, contradictory to research undertaken by Young et al. [61], who found higher satisfaction was correlated with higher fidelity and thus exposure to the intervention. Moreover, teachers praised the intervention in regards to how the intervention was integrated into the timetable and had strong cross-curricular links; a practice recommended by the UK government in the delivery of health promotion topics [62].

Despite the positive overview of SFS given, modifications were recommended for future delivery. In regards to the training workshop and manual (including session plans), predominately, modifications surrounded aiding deliverer’ self-efficacy in regards to the delivery of sessions, as well as increasing children’s engagement in sessions and understanding of smoking related messages. Recommendations included having more time to practice delivery during the practical section of the bespoke training, improving the user-ability of the manual through the inclusion of visual diagrams and/or DVD, and modifying games to reduce sedentary time during sessions. Addressing sedentary time during sessions is important since the philosophical underpinning of the intervention was to deliver smoking-related messages through physical activity. Notably, sedentary periods during coaching sessions often related to barriers surrounding class/hall size and modifications made by coaches to session plans. Data suggests that whilst flexibility should be built into the session plans to allow for differences in settings across schools, the importance of intervention fidelity in regards to core components must be reinforced to deliverers, ensuring time spent sedentary during activities is minimised.

Whilst coaching sessions were considered educational, teachers noted smoking-related messages delivered were on occasions inaccurate. Notably, direct observations of coaching sessions confirmed that messages were not always delivered as outlined in the manual. Teachers recommended coaches had a full understanding of messages before delivery of sessions. Whilst previous research has shown coaches can be trained to deliver smoking prevention messages through sport [21], a review of school-based drug abuse prevention interventions documented extensive training, including follow-ups, was associated with higher quality implementation and outcomes [44]. Therefore, on-going training and monitoring for deliverers as well as formative feedback and consultation during early phases of delivery should be considered, as found previously [63].

Overall, advantages of utilising either coaches or teachers to deliver SFS were recognised, as well as the simultaneous employment of both. Whilst the benefits of using both teachers and coaches to deliver SFS included teachers learning from coaches, it was noted teachers often did not engage fully in SFS coaching sessions. Research has highlighted the benefits of observing coaching and participating in sessions enhances teachers’ skills and confidence in regards to their ability to effectively deliver PE [64], and further methods to engage teachers in SFS coaching sessions are needed. Increasing teachers’ skills and confidence in leading SFS session may lead to higher levels of intervention implementation [38; Garnham-Lee et al., unpublished data], with wider implications for sport-for-health intervention generally.

The importance of sustaining the intervention within the school setting was recognised. Sustainability was discussed in regards to maintaining intervention effectiveness and increasing school awareness of SFS. In regards to maintaining perceived intervention effectiveness, it is recognised interventions focusing on the individual often require multiple exposures [65]. Since teachers reported the programme to be valuable and would recommend it to others, this would also be indicative of willingness to continue engagement, particularly with core components where attrition rates were low. Increasing school wide awareness of the intervention, for example through school policy, may also aid teacher engagement and intervention dose [66].

Whilst insights have been gained into the implementation of the intervention, a number of limitations and implications for future research are recognised. Self-report data to ascertain intervention implementation is commonly used in school-based health promotion studies, but may overestimate actual dose [44] and researchers may lack agreement with teachers’ fidelity scores if direct observation had taken place (see [67]). Direct observations of a sub-sample of SFS sessions delivered by teachers to assess researcher-teacher agreement of fidelity scores would have been beneficial. Video recording SFS coaching sessions would have also allowed for researchers to cross-check scoring of fidelity aiding reliability of data. Moreover, some children, teachers and coaches were unable to explain in detail intervention components during post-data collection. Whilst self-evaluations of teachers’ sessions were completed throughout the intervention it would have been advantageous to explore participants’ perceptions of all intervention components during the study period, similar to the ASSIST intervention [68]. To provide teachers who did not engage fully with the intervention with the necessary support to lead SFS sessions, a comparative study of teachers who delivered sessions and those who did not would have been beneficial, and a recommendation for future research. Finally, the intervention was delivered in deprived neighbourhoods within a single large urban city in North West England, limiting the generalisability of findings. Acceptability of the intervention across different populations, where cultural values surrounding smoking and participation in sport may differ, needs to be investigated.

## Conclusion

This process evaluation explored the reach, dose, fidelity, acceptability and sustainability of SFS and provides useful information regarding the feasibility and implementation of a novel sport-for-health intervention. Overall, SFS was considered acceptable to children, teachers and coaches. Nevertheless, efforts to enhance intervention reach (at the school level), dose (teachers’ engagement) and sustainability must be considered. Variations in dose and fidelity likely reflect challenges associated with delivery of a complex intervention within school settings. It is suggested greater flexibility must be built into the delivery of intervention components to ensure fidelity of intervention implementation is not compromised. Increasing school awareness of the intervention may subsequently increase its dose and sustainability. How variations in dose and fidelity will impact on intervention effectiveness will be inferred from impact data. If proven to have a long-term positive impact on children’s smoking-related cognitions, there will be grounds to promote sport as an important component of a smoking prevention strategy.

## Abbreviations

PE: Physical education
PSHE: Personal social health and economic
SFS: SmokeFree Sports
